# The WHO-5 Well-Being Index: A Validation Study in People with Infertility

**Published:** 2019-11

**Authors:** Reza OMANI-SAMANI, Saman MAROUFIZADEH, Amir ALMASI-HASHIANI, Mahdi SEPIDARKISH, Payam AMINI

**Affiliations:** 1. Department of Medical Ethics and Law, Reproductive Biomedicine Research Center, Royan Institute for Reproductive Biomedicine, ACECR, Tehran, Iran; 2. School of Nursing and Midwifery, Guilan University of Medical Sciences, Rasht, Iran; 3. Department of Epidemiology, School of Health, Arak University of Medical Sciences, Arak, Iran; 4. Department of Biostatistics and Epidemiology, Babol University of Medical Sciences, Babol, Iran; 5. Department of Biostatistics and Epidemiology, School of Public Health, Ahvaz Jundishapur University of Medical Sciences, Ahvaz, Iran

**Keywords:** Infertility, WHO-5, Reliability, Validity, Iran

## Abstract

**Background:**

Infertility is a public health problem and can lead to depressive symptoms. In recent years, the WHO-five Well-being Index (WHO-5) has been used as a screening measure for depression, but study on psychometric properties in people with infertility is scarce. The objective of this study was to examine the reliability and validity of the Persian version of the WHO-5 in people with infertility.

**Methods:**

Overall, 539 infertile patients from a referral infertility center in Tehran, Iran in the period between May and Aug 2017, completed the WHO-5, along with other psychological measures: the Patient Health Questionnaire-9 (PHQ-9) and the Hospital Anxiety and Depression Scale (HADS). Construct validity and internal consistency of WHO-5 were evaluated using confirmatory factor analysis (CFA) and Cronbach’s alpha, respectively. Convergent validity was examined by relationship with PHQ-9 and HADS.

**Results:**

The prevalence of poor well-being was 44.3% and that of depression was 18.6%. CFA confirmed the unidimensional factor structure of the WHO-5. Internal consistency of the WHO-5 was good (Cronbach’s alpha=0.858). The WHO-5 significantly correlated with the PHQ-9 (r=–0.522), HADS-anxiety (r=–0.524) and HADS-depression (r=–0.630), confirming convergent validity.

**Conclusion:**

The WHO-5 is a short and easy to use questionnaire with satisfactory reliability and validity that appears suitable for use as a screening test for depressive symptom in infertile people. In addition, the prevalence of depression and poor well-being was very high in this population.

## Introduction

Infertility is defined by “the failure to establish a clinical pregnancy after 12 months of regular, unprotected sexual intercourse or due to an impairment of a person's capacity to reproduce either as an individual or with his/her partner.” (1), and affects approximately 9% of reproductive-aged couples throughout the world (2). In Iran, the overall prevalence of infertility was 13.2% (3). Infertility, besides being a medical problem, is a psychosocial condition.

It is a severe stressor in life with negative psychological consequences. Among these consequences, depression is one of the most common psychiatric disorders and adversely affects quality of life and infertility outcomes (4–6). Infertile people also experience more depressive symptoms compared to general population (7). General-population surveys on major depression in Europe and the USA yielded a 1-year prevalence of 5.7%, 6.7% respectively (8, 9). Previous studies in infertile people yielded a prevalence of 36.7% in the USA (10), 35.4% in Poland (11), 33.3% in Iran (7), and 31.0% in Pakistan (12). Epidemiological studies show that increased risk for depression is associated with being female, low educational level, long infertility duration and failure in previous treatment (7, 13, 14). Screening for depressive symptoms be performed routinely in this population, but there is no consensus on which measure to use for this purpose. Numerous valid and reliable instruments are currently available to measure depression.

One particularly popular measure is the WHO-5 Well-Being Index (WHO-5). The WHO-5 is a short, self-administered, and positively worded scale designed to measure the level of subjective well-being over the last two weeks (15, 16). Although the WHO-5 was originally developed as a measure of subjective well-being, numerous studies suggest that it also has satisfactory psycho-metric properties for assessing depressive symptoms.

Although the WHO-5 instrument has demonstrated satisfactory psychometric properties in various clinical and non-clinical samples (17–23), it is yet to be examined in people with infertility. We, therefore, performed this study to examine the reliability and validity of the WHO-5 in a sample of people with infertility.

## Materials and Methods

### Participants and Study Design

In this cross-sectional study, data were collected from 539 infertile patients attending at Royan Institute, Tehran, Iran in the period between May and Aug 2017. To be eligible for this study, participants had infertility problem; be 18 yr of age or older, and be able to read and write in Persian.

Ethical approval to conduct the present study was granted by the Ethics Committee of Royan Institute, Tehran, Iran. All patients were fully informed about the aim of the study and the confidentiality of the data. Prior to data collection, written informed consent was obtained from each participant.

### Questionnaires

The questionnaire included demographic/clinical factors, WHO-5, Patient Health Questionnaire-9 (PHQ-9), and Hospital Anxiety and Depression Scale (HADS).

### Demographic/Clinical Characteristics

Demograpic and clinical characteristics, including age, gender, educational level, duration of infertility, cause of infertility, failure of previous treatment, and history of abortion were collected.

### WHO-5 Well-Being Index (WHO-5)

The WHO-5 is a short, self-administered measure of well-being over the last two weeks (15, 16). It consists of five positively worded items that are rated on 6-point Likert scale, ranging from 0 (at no the time) to 5 (all of the time). The raw scores are transformed to a score from 0 to 100, with lower scores indicating worse well-being. A score of ≤50 indicates poor wellbeing and suggests further investigation into possible symptoms of depression. A score of 28 or below is indicative of depression. The Persian version of WHO-5 available at ( https://www.psykiatri-regionh.dk/who-5/Pages/default.aspx ).

### Patient Health Questionnaire-9 (PHQ-9)

The PHQ-9 is a 9-item self-report tool designed to assess depression severity (24). The items duplicate the nine diagnostic criteria for major depressive disorder covered in the Diagnostic and Statistical Manual of Mental Disorders, Fourth Edition (DSM-IV). The PHQ-9 asks how often participants have been bothered by problems in the past 2 wk. Each item scored on a 4-point Likert scale, ranging from 0 (not at all) to 3 (nearly every day). The PHQ-9 total score can range from 0–27, with a score of ≥10 is indicative of depression. The PHQ-9 showed high internal consistency in this study, with a Cronbach’s alpha of 0.851.

### Hospital Anxiety and Depression Scale (HADS)

The HADS is a 14-item self-administered measure of anxiety (HADS-A, 7 items) and depression (HADS-D, 7 items) disorders (25). Each item is rated on a 4-point Likert scale, ranging from 0 to 3. Subscale scores can range from 0 to 21, where higher scores indicate greater level of anxiety and depression. The Persian version of HADS has been validated and frequently used in infertile patients (26). In the current study, both HADS-A and HADS-D had good internal consistency, with a Cronbach’s alpha of 0.884 and 0.783, respectively.

#### Statistical Analysis

The CFA using maximum likelihood estimation method was conducted in order to examine the one-factor structure of WHO-5. Model fit was assessed using the following criteria: the chi-square/degree of freedom (χ^2^/df), the comparative fit index (CFI), the root mean square error of approximation (RMSEA), and the standardized root mean square residual (SRMR). Model fit was interpreted as ‘acceptable’ if χ^2^/df<3, CFI>0.9, RMSEA<0.08, and SRMR<0.08 (for good fit: χ^2^/df<2, CFI>0.95, RMSEA<0.06, and SRMR<0.05) (27, 28). Internal consistency of the scale was investigated by computing (a) Cronbach’s alpha, (c) inter-item correlation, and (c) corrected-item total correlation. Finally, convergent validity will be examined by computing the relations among the WHO-5 total score and measures of HADS and PHQ-9. Statistical analyses were done with IBM SPSS Statistics for Windows, ver. 22.0 (IBM Corp., Armonk, NY, USA) and Lisrel 8.80 (Scientific Software International, Inc., Lincolnwood, IL, USA).

## Results

### Participant Characteristics

Table 1 outlines the demographic/fertility characteristics of the 539 participants (249 men and 290 women). The mean age and infertility duration of the study sample were 32.97 (SD: 5.34) and 5.55 (SD: 4.07) yr, respectively. The majority of participants were male factor (41.4%), 50.4% were university-educated, 53.1% had no failure in previous treatments and 70.9% had no history of abortion.

**Table 1:** Demographic and clinical characteristics of the participants (n=539)

***Variable***	***Mean ± SD or n (%)***
Age (yr)	32.97 ± 5.34
Sex	
Male	249 (46.2)
Female	290 (53.8)
Educational level	
Primary	92 (17.1)
Secondary	175 (32.5)
University	272 (50.4)
Duration of infertility (years)	5.55 ± 4.07
Cause of infertility	
Male factor	223 (41.4)
Female factor	95 (17.6)
Both	112 (20.8)
Unexplained	109 (20.2)
Failure of previous treatment	
No	253 (46.9)
Yes	286 (53.1)
History of abortion	
No	382 (70.9)
Yes	157 (29.1)

SD: Standard deviation

### Descriptive Statistics of WHO-5

Item wording means, and standard deviation for WHO-5 are presented in Table 2. The item means ranged from 2.44 (for item “My daily life has been filled with things that interest me”) to 2.86 (for item “I have felt cheerful and in good spirits.”). The mean WHO-5 total score was 53.70 ± 23.45 (range, 0-100). The prevalence of poor well-being (WHO-5 score≤50) was 44.3% (n=239) and that of depression (WHO-5 score≤28) was 18.6% (n=100).

**Table 2:** Items wording and descriptive statistics, and internal consistency of the WHO-5

	***Variable***	***Mean***	***SD***	***Corrected item total correlation***	***Alpha Cronbach’s if item deleted***	***Alpha***
1	I have felt cheerful and in good spirits.	2.86	1.38	0.682	0.827	
2	I have felt calm and relaxed.	2.78	1.41	0.718	0.818	
3	I have felt active and vigorous.	2.82	1.48	0.698	0.823	
4	I woke up feeling fresh and rested.	2.52	1.55	0.643	0.838	
5	My daily life has been filled with things that interest me.	2.44	1.51	0.636	0.839	
	WHO-5 Total Score	53.70	23.45			0.858

SD: Standard deviation

### Internal Consistency

The WHO-5 showed good internal consistency with Cronbach’s alpha of 0.858, and this value did not improve if an item was deleted from the scale. The corrected item-total correlations ranged from 0.636 to 0.718 with a mean of 0.675. The inter-item correlations among the WHO-5 items were high, ranging from 0.486 to 0.680.

### Confirmatory Factor Analysis

The CFA was used for testing the unidimensionality of the WHO-5.

**Fig. 1:**
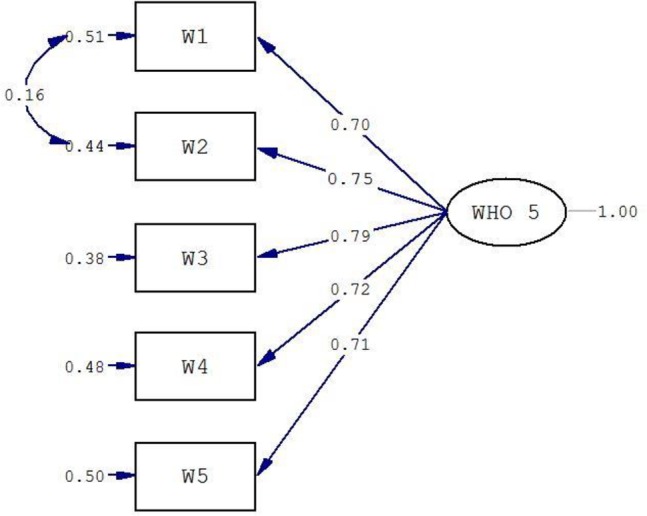
Confirmatory factor analysis of the one-factor model WHO-5

The fit of the model was not good based on the fit indices (χ^2^/df=8.12; CFI=0.98; RMSEA=0.115 and SRMR=0.031). Examination of the modification indices recommended allowing covariance between Item 1 and Item 2 (Fig. 1). A superior fit was obtained after allowing for this covariance (χ^2^/df=1.11; CFI=0.99; RMSEA=0.014 and SRMR=0.010).

### Convergent Validity

Convergent validity of the WHO-5 was confirmed by its considerable correlations with other relevant scales: HADS-A (r=–0.524, *P*<0.001), HADS-D (r=–0.630, *P*<0.001), and PHQ-9 (r=–0.522, *P*<0.001). In addition, comparison indicated that the correlation between WHO-5 and HADS-D was significantly stronger than the correlation between WHO-5 and HADS-A (z=2.61, *P*=0.009).

## Discussion

The current study examined the psychometric properties of the WHO-5 in a sample of patients with infertility. These patients experience more depressive symptoms compared to general population (7) and had poor quality of life and life satisfaction (4, 29). Although the WHO-5 questionnaire has demonstrated satisfactory psychometric properties in general population (17, 18), patients with diabetes (19–22), and primary care (23), it is yet to be examined in a population with infertility. In the present study, the mean WHO-5 score was 53.70 ± 23.45, which is lower than what was reported in a general population (64.74 ± 18.80) (17). Furthermore, the prevalence of poor well-being and depression in this study was 44.3% and 18.6%, respectively, which is higher than what was reported in general population (8, 9, 30). In this study, a lower prevalence was found than other studies conducted among infertile patients and this is probably due to the different tools used to examine depression (7, 10–12). The unidimensional structure of the WHO-5 reported in previous works (31–33) was confirmed in this study. The initial CFA analysis did not produce an acceptable fit, however, by allowing covariance between item 1 (I have felt cheerful and in good spirits) and item 1 (I have felt calm and relaxed) we obtained a superior fit. This makes sense conceptually as "feeling calm and relaxing" and "feeling cheerful and in good spirit" are closely connected especially in this population.

The internal consistency of the WHO-5 was high. Furthermore, the corrected item-total correlations, as well as the inter-item correlations, were also within acceptable range. These findings are in line with what was reported in previous studies in different populations (17–23). Support for the convergent validity of the scale was evidenced. That is, we found that the WHO-5 scores were significantly correlated with HADS-anxiety, HADS-depression, and PHQ-9. This finding is compatible with previous studies showed that WHO-5 score is correlated with measures of depression, anxiety, stress, well-being, mental health and self-esteem, quality of life. Several limitations of the present study should be noted. First, it was a single-center study, thus, the generalization of the results may be limited. Second, diagnostic interviews Structured Clinical Interview for DSM or another clinical interview were not conducted, precluding any discussion of the sensitivity and specificity the scale. Third, the test-retest reliability of the scale was not evaluated in this study.

## Conclusion

The Persian version of WHO-5 has adequate psychometric properties and support its use as a screening instrument for depressive symptom in infertile people. Furthermore, its brevity and ease of use makes it a potentially suitable instrument for identify subjects with depressive symptom in large epidemiological studies. In addition, the prevalence of depression and poor well-being was very high in infertile patients; therefore, a holistic approach, including psychological interventions and support, is absolutely essential to reduce depression symptoms in this population.

## Ethical considerations

Ethical issues (Including plagiarism, informed consent, misconduct, data fabrication and/or falsification, double publication and/or submission, redundancy, etc.) have been completely observed by the authors.
